# Successful laparoscopic resection of ovarian abscess caused by *Staphylococcus aureus* in a 13-year-old girl: a case report and review of literature

**DOI:** 10.1186/s12905-021-01335-z

**Published:** 2021-05-13

**Authors:** Tsuyoshi Murata, Yuta Endo, Shigenori Furukawa, Atsushi Ono, Yuichiroh Kiko, Shu Soeda, Takafumi Watanabe, Toshifumi Takahashi, Keiya Fujimori

**Affiliations:** 1 Department of Obstetrics and Gynecology, Fukushima Medical University School of Medicine, Fukushima, 960-1295 Japan; 2 Department of Pediatrics, Fukushima Medical University School of Medicine, Fukushima, 960-1295 Japan; 3 Department of Diagnostic Pathology, Fukushima Medical University School of Medicine, Fukushima, 960-1295 Japan; 4 Fukushima Medical Center for Children and Women, Fukushima Medical University, Fukushima, 960-1295 Japan

**Keywords:** Ovarian abscess, Pelvic inflammatory disease, *Staphylococcus aureus*, Virginal girl, Case report

## Abstract

**Background:**

Ovarian abscesses, which occur mostly in sexually active women via recurrent salpingitis, occur rarely in virginal adolescent girls. Here, we present a case of an ovarian abscess in a virginal adolescent girl who was diagnosed and treated by laparoscopy.

**Case presentation:**

A 13-year-old healthy girl presented with fever lasting for a month without abdominal pain. Computed tomography scan and magnetic resonance imaging indicated a right ovarian abscess. Laparoscopic surgery revealed a right ovarian abscess with intact uterus and fallopian tubes. The abscess was caused by *Staphylococcus aureus*. The patient recovered completely after excision of the abscess, followed by antibiotic treatment.

**Conclusions:**

Ovarian abscess may occur in virginal adolescent girls; *Staphylococcus aureus*, an uncommon species causing ovarian abscess, may cause the infection.

## Background

Ovarian abscess (OA), a form of tubo-ovarian abscess (TOA), is a complication of pelvic inflammatory disease (PID), which most often occurs in sexually active women via recurrent salpingitis [1]. Hence, OA is rare in virginal adolescent girls [2]. Since torsion of the OA, rupture leading to sepsis, and infertility may occur, quick diagnosis and laparoscopic treatment is strongly recommended [1, 2]. Here, we present a case of OA in a virginal adolescent girl who was treated by laparoscopy.

## Case presentation

A 13-year-old healthy virginal adolescent girl was referred to the university hospital by a local doctor after she presented with persistent fever (up to 39 °C for a month) without any other symptoms. Laboratory data showed a white blood cell (WBC) count of over 10.0 × 10^3^/µL and a C-reactive protein (CRP) level of approximately 7.0 mg/dL. The patient had been treated with oral amoxicillin and tosufloxacin for 2 weeks based on a suspected bacterial infection; however, the blood cultures performed on the day of referral to the university hospital were negative.

Besides a body temperature of 37.4 °C, physical examination by the pediatric physician in the university hospital revealed no abnormalities. The patient had no history of dental treatment, trauma, or cystitis. Laboratory data showed a WBC count of 8.1 × 10^3^/µL, a hemoglobin level of 11.5 g/dL, a platelet count of 29.5 × 10^3^/µL, and a CRP level of 6.4 mg/dL. Additionally, assays for soluble interleukin-2 receptor and autoantibodies, indicative of malignant lymphoma and collagen disease, respectively, were negative. Serological tests for human immunodeficiency virus and tuberculosis were also negative. The rapid antigen test for group A streptococci and polymerase chain reaction test for coronavirus disease 2019 were also negative.

Trans-abdominal ultrasound showed a pelvic mass measuring 6 cm; therefore, gynecologists were consulted. Menarche had occurred at the age of 12 years. The menstrual cycle was irregular, and the development of breast and pubic hair was in Tanner stage II. The patient had a height of 150 cm and body weight of 35 kg. The patient had no history of sexual activity, sexual abuse, or transvaginal maneuver; therefore, bimanual examination was not applicable to this patient.

**Fig. 1:**
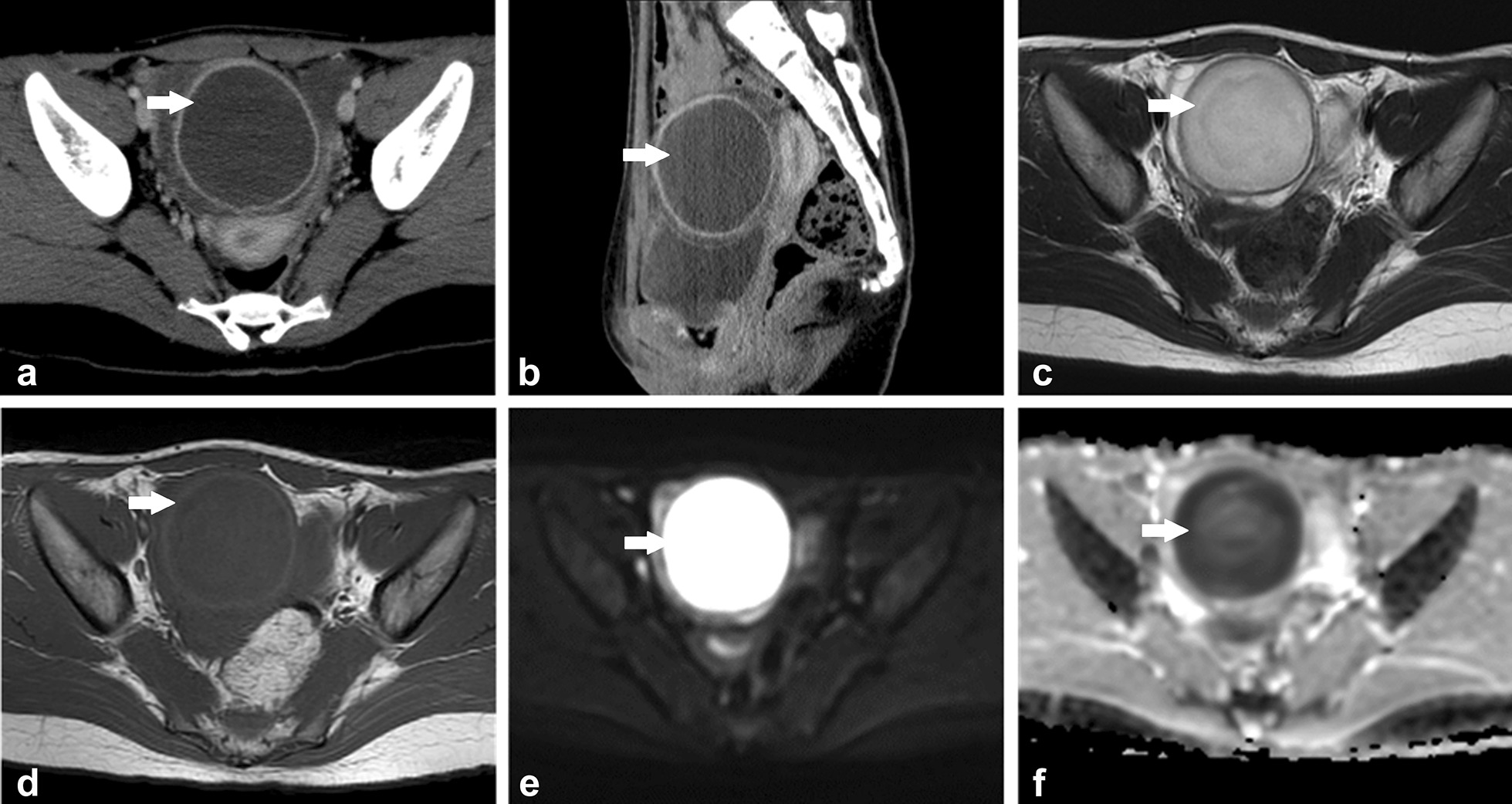
Computed tomography scan shows a unilateral and unilocular ovarian mass with dense fluid and a thick, uniform, enhancing wall in a transverse section image (**a**) and a sagittal image (**b**). Magnetic resonance imaging shows a right ovarian mass with intermediate signal intensity on T2-weighted image (**c**), low signal intensity on T1-weighted image (**d**), high signal intensity on diffusion-weighted image (**e**), and low diffusion, indicated by the apparent diffusion coefficient (**f**). The white arrow shows the abscess

Computed tomography (CT) scan was performed initially to evaluate the blood supply into the ovarian tumor and evaluate for other sources of fever. The scan revealed a unilateral and unilocular ovarian mass with a thick, uniform, enhancing wall. Additionally, the fluid in the mass was dense. These observations suggested the presence of OA (Fig. 1a, b). Pyosalpinx was not confirmed. CT scan revealed no other possible origin of fever. Magnetic resonance imaging (MRI) revealed intermediate signal intensity on T2-weighted images, low signal intensity on T1-weighted images, high signal intensity on diffusion-weighted imaging (DWI), and the apparent diffusion coefficient (ADC) indicated low diffusion. These observations also indicated the presence of OA (Fig. 1c–f).

**Fig. 2:**
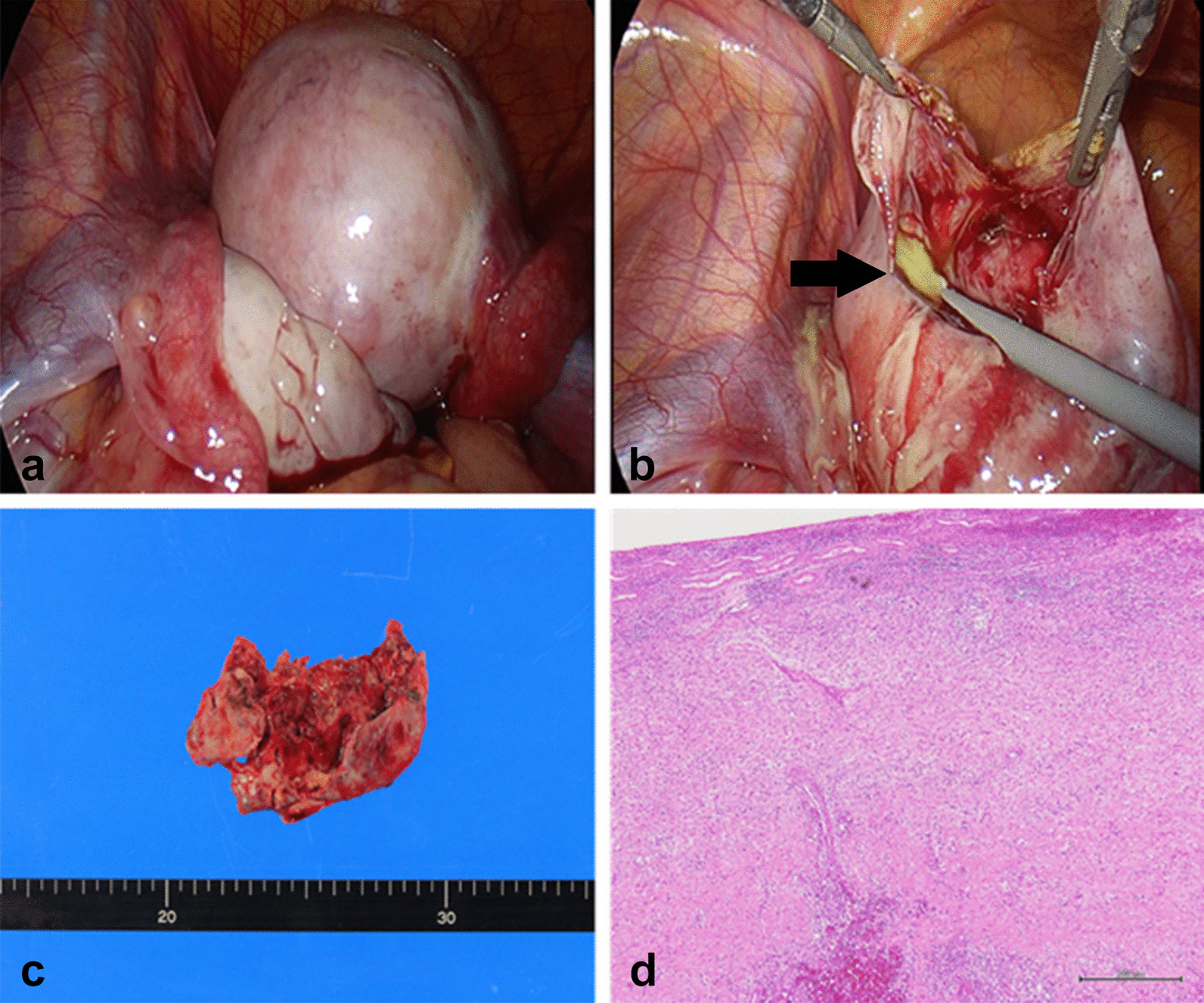
Laparoscopy shows the right ovarian abscess with intact fallopian tube and intact left ovary with intact fallopian tube before excision (**a**) and during excision (**b**, **c**). The black arrow shows the abscess. The histological findings revealed granulation tissue with neutrophilic infiltration, suggesting abscess formation in ovary, without indication of basal ovarian tumor (scale bar: 500 µm) (**d**)

Taking into consideration the risk of ovarian torsion and acute sepsis due to persistent fever, laparoscopic surgery, involving four incisions to insert four ports, was performed for confirmation and excision of the abscess. Laparoscopy revealed a right ovary, swollen by 5 cm, with an intact fallopian tube (Fig. 2a); an intact left ovary with intact fallopian tube; and a small amount of ascites. Puncturing of the swollen right ovary revealed internal pus, which confirmed the diagnosis of OA. The pus was collected for bacterial culture and the abscess was excised without any substantial compromise to the ovary (Fig. 2b, c). We performed pelvic washing. The postoperative laboratory data 3 days after surgery showed a CRP level of 13.8 mg/dL. The patient received intravenous cefmetazole for 5 days at a dose of 2 g/day to prevent recurrence of the infection. The postoperative course was uneventful, and the patient was discharged 6 days after surgery; the CRP level on the day of discharge was 4.12 mg/dL. The bacterial culture of the abscess showed the presence of methicillin-susceptible *Staphylococcus aureus*. Oral cefaclor at a dose of 900 mg/day was continued for 14 days after intravenous cefmetazole. The histological findings revealed granulation tissue with neutrophilic infiltration, suggesting abscess formation in the ovary, without indication of basal ovarian tumor (Fig. 2d). Malignancy was not observed. Finally, CRP level was within the normal range, and MRI performed 1 month after surgery showed no recurrence of OA.

This study did not require ethics approval by the institutional review board at Fukushima Medical University. Written informed consent was obtained from the patient and her mother for the publication of the report.

## Discussion and conclusions

The present case demonstrates that OA can occur in a healthy virginal adolescent girl. Literature review (up to 2019) identified 18 cases of virginal girls and women with TOA or OA (Table 1) [2–7], which suggests that OA occurs rarely in virginal girls. The cause of TOA in this patient group is often unclear; however, virginal girls have been speculated to have comorbidities, such as vaginal voiding causing ascending infection, gastrointestinal tract translocation, congenital genitourinary anomalies, previous pelvic surgery, and bacteremia from skin wounds, which predispose them to TOA [1, 2]. Physicians should therefore consider the possibility of TOA in virginal girls presenting with fever.

**Table 1:** Review of the tubo-ovarian abscess cases reported to date in virginal girls and women. (Revised from Cho et al. [2])

Case No.	Authors	Year of case publication	Age (years)	Symptoms	Preoperative diagnosis	Postoperative diagnosis	Surgical procedure	Concomitant events as possible causal factors	Species
1	Teng et al.	1996	47	Abdominal pain, fever	TOA or peri-appendiceal abscess	TOA	Hysterectomy, BSO, appendectomy	Bacteremia after cat scratch	*Pasteurella multocida*
2	Moore et al.	1999	15	Abdominal pain, fever	Pelvic mass	TOA	LSO	Recurrent UTI	*Escherichia coli*
3	Leong and Bowditch	2001	23	Abdominal pain	Ovarian tumor	TOA	Laparoscopic RSO	Unknown	Negative
4	Fumino et al.	2002	13	Abdominal pain, fever	TOA	TOA	LSO	Vaginoplasty for cloacal anomaly	Not mentioned
5	Dogan et al.	2004	19	Abdominal pain	Ovarian tumor	TOA	Wedge resection of ovary	Ascending infection from the lower genital tract	*Escherichia coli*, etc
6	Arda et al.	2004	15	Abdominal pain, fever	TOA	TOA	Laparoscopic abscess drainage	Concomitant UTI	*Escherichia coli*
7	Hartmann et al.	2009	16	Abdominal pain, fever	Inflammation of the ovary	TOA	Laparoscopy for diagnosis	Crohn’s disease	*Bacteroides uniformis*, etc
8	Hartmann et al.	2009	12	Abdominal pain, fever	Large dominant ovarian cyst	TOA	Laparoscopy for diagnosis	Recurrent UTI	*Escherichia coli*
9	Gensheimer et al.	2010	20	Abdominal pain, fever	Complex hemorrhagic cyst	TOA	RSO, small bowel resection, appendectomy	Genitourinary or gastrointestinal spread	*Abiotrophia* spp., etc
10	Ashrafganjooei et al.	2011	24	Abdominal pain, fever	Necrotic pelvic tumor or pelvic abscess	TOA	TAH, RSO	Unknown	Mixed organisms
11	Tuncer et al.	2012	30	Abdominal pain, fever	TOA	TOA	Percutaneous drainage	Sigmoid diverticulitis	*Escherichia coli*, etc
12	Sakar et al.	2012	13	Abdominal pain, menstrual disorder	Ovarian tumor	TOA	Left salpingectomy	Ascending infection from the lower genital tract	Not mentioned
13	Simpson-Camp et al.	2012	14	Abdominal pain, fever	Borderline mucinous tumor	TOA	Laparotomy for diagnosis	Ascending infection from the lower genital tract	*Streptococcus viridans*
14	Goodwin et al.	2013	13	Abdominal pain	Bowel compromise	TOA	Abscess drainage	Bacterial bowel translocation	*Escherichia coli*
15	Cho et al.	2017	21	Abdominal pain, fever	Hemorrhagic corpus luteal cyst	OA	Abscess drainage, appendectomy	Unknown	Negative
16	Alsahabi et al.	2017	19	Abdominal pain, fever	Tubo-ovarian malignant tumor	TOA	Percutaneous drainage	Unknown	*Escherichia coli*, etc
17	Stortini et al.	2017	14	Acute urinary retention	TOA	TOA	Percutaneous drainage	Obstructive lesions in the genital tract due to labial agglutination	*Streptococcus anginosus,* etc
18	Mills et al.	2018	13	Abdominal pain, fever	TOA	TOA	Laparoscopic abscess drainage	Bacterial translocation secondary to chronic appendicitis	*Streptococcus constellatus*
19	Murata et al. (present case)	2021	13	Fever	OA	OA	Laparoscopic abscess resection	Unknown	*Staphylococcus aureus*

*BSO* bilateral salpingo-oophorectomy, *LSO* left salpingo-oophorectomy, *OA* ovarian abscess, *RSO* right salpingo-oophorectomy, *TAH* total abdominal hysterectomy, *TOA* tubo-ovarian abscess, *UTI* urinary tract infection

Quick diagnosis of OA is desired because it increases the risk of torsion and sepsis. When OA is suspected, quick treatment is required to prevent adverse outcomes [1, 2]. In the present case, laparoscopic surgery was selected due to  an increased risk of torsion and sepsis; the fever had persisted despite antibiotics treatment for two weeks. However, the rarity and uncommon clinical symptoms of OA in virginal girls may lead to misdiagnosis (Table 1). CT scan and MRI may help in the preoperative diagnosis of OA in virginal adolescent girls. The majority of TOA cases have been reported to be unilateral (73%) and multilocular (89%) with the presence of dense fluid (95%); a thick, uniform, enhancing wall (95%); thickening of the mesosalpinx (91%); pelvic fat infiltration (91%); thickening of the uterosacral ligaments (64%); and pyosalpinx (50%) [8]. Additionally, TOA has been reported to show low or intermediate signal intensity on T2-weighted images, high signal intensity on DWI, and low diffusion on ADC in MRI [9]. Notably, MRI with DWI has been reported to have higher accuracy than CT scan and MRI without DWI [9]. Therefore, physicians should evaluate CT scan and MRI for preoperative diagnosis of an ovarian mass in virginal girls, as was done in the present case.

To the best of our knowledge, the present case is the first case of an OA caused by *S. aureus* in a virginal girl (Table 1). PID, including TOA, is commonly caused by ascending genital infections (*Escherichia coli* and *Enterococcus* spp) [2]. However, the patient in the present case was sexually inactive, and OA was caused by *S. aureus*, which has been rarely known to cause TOA. To date, only two cases of TOA caused by *S. aureus* in non-virginal adult women have been reported; one was an acute TOA after intrauterine device insertion and the other was after tubal ligation [10]. In the present case, since the fallopian tubes were intact and the patient had no episode of transvaginal maneuver, the source of the infection could have been via the bloodstream. *S. aureus* is one of the most common gram-positive bacteria responsible for sepsis. Moreover, the patient had no history of dental treatment, trauma, or compromised immune system; therefore, no alternative source of infection was identified. The present case suggests that physicians should consider the possibility of uncommon bacterial species as the causative agents for OA among virginal girls and that these species may cause infection from an unknown origin via the bloodstream.

In conclusion, OA may occur in virginal adolescent girls. *S. aureus*, a rare species causing OA, may be the underlying cause of infection via the bloodstream. Physicians should consider the possibility of OA among virginal girls and should carefully evaluate for underlying causes, including the bacterial species and the possible source of bacterial infection.

## Data Availability

All data generated or analyzed during this study are included in this published article, as this is a case report.

## Abbreviations

ADC: Apparent diffusion coefficient
CRP: C-reactive protein
CT: Computed tomography
DWI: Diffusion-weighted imaging
MRI: Magnetic resonance imaging
OA: Ovarian abscess
PID: Pelvic inflammatory disease
TOA: Tubo-ovarian abscess
WBC: White blood cell
